# The impact of teacher support on self-regulated learning in piano instruction at teacher-training universities: the mediating role of self-efficacy and the moderating role of mastery goals

**DOI:** 10.3389/fpsyg.2026.1743188

**Published:** 2026-01-15

**Authors:** Jieyuan Li

**Affiliations:** School of Music, Hefei Normal University, Hefei, Anhui, China

**Keywords:** mastery goals, piano instruction, self-efficacy, self-regulated learning, teacher support

## Abstract

**Objective:**

This study examines examined the association between teacher support and students’ self-regulated learning (SRL) in piano instruction, investigating the mediating role of self-efficacy and the moderating role of mastery goals.

**Methods:**

Data were collected from 464 music education majors at teacher-training universities in China using adapted and validated scales measuring teacher support, self-efficacy, mastery goals, and SRL. The hypothesized model was evaluated for overall fit using structural equation modeling (SEM). The specific mediation and moderated effects were then tested via regression-based path analysis using the PROCESS macro with the bootstrap method.

**Results:**

Teacher support was significantly and positively associated with students’ self-regulated learning in piano (total effect = 0.49). Self-efficacy partially mediated this relationship, accounting for a considerable portion (70.9%) of the total effect. Furthermore, mastery goals significantly moderated both the effect of teacher support on self-efficacy and its direct effect on self-regulated learning.

**Conclusion:**

These findings provide a theoretical foundation and practical implications for constructing a multidimensional teacher support system, enhancing students’ self-efficacy, and fostering a value orientation toward mastery goals in piano pedagogy within teacher-training institutions.

## 1 Introduction

Music teacher education in teacher-training universities in China (historically and administratively known as “Normal Universities”) carries the significant responsibility of cultivating future music educators. Its core objectives extend beyond the transmission of proficient piano skills to encompass the shaping of artistic character and the cultivation of lifelong learning capabilities (Lehmann et al., 2007). Within the piano curriculum of such programs, this dual imperative is often emphasized: students are expected not only to achieve technical mastery but also to develop the pedagogical insight and self-directed learning capacities they will later need to foster in their own students (Lehmann et al., 2007). As a pivotal component of the music education curriculum, piano instruction is widely regarded as a key factor influencing the quality of music professional training (Tong, 2008; Wang, 2024). However, piano pedagogy in this specific teacher-training context faces unique challenges that necessitate a deeper investigation into its motivational dynamics, distinguishing it from training in conservatories focused primarily on performance excellence. Traditional piano pedagogy, even within higher education, has predominantly emphasized rigorous technical training and teacher-directed correction, an approach that a growing body of research suggests may be insufficient for fostering the adaptive, self-sustaining learners required in modern education (Niemiec and Ryan, 2009; Panadero, 2017; Puustinen and Pulkkinen, 2001). Contemporary university students, including those in music programs, commonly encounter multifaceted challenges in skill-based learning, such as those related to self-regulation, competence development, and future career adaptation (Jääskelä et al., 2017). Within the specific context of piano studies for music education majors, these general challenges manifest in domain-specific forms, including difficulties in internalizing high-precision techniques, cultivating artistic expressiveness, and navigating the transition from student to educator. An examination of these challenges through the lens of Self-Determination Theory (Ryan and Deci, 2020) suggests that an instructional approach overly reliant on external control and technical discipline may be limited. Such an approach risks undermining students’ basic psychological needs for autonomy, competence, and relatedness, which are essential for fostering the intrinsic motivation and deep, self-regulated learning that are increasingly recognized as critical objectives for cultivating adaptable musicians and educators in modern higher music education (McPherson and Zimmerman, 2011). Consequently, effectively stimulating students’ intrinsic motivational mechanisms, particularly fostering their core capacity for self-regulated learning, has become a crucial focal point for overcoming current bottlenecks in instructional efficacy and enhancing the quality of future music educator training (Zimmerman, 2002).

The classroom serves as the primary context for piano instruction, and its quality is highly dependent on positive interactions between teaching and learning. Within this dynamic, teacher support (e.g., emotional care, competence feedback, autonomy stimulation) can be conceptualized as a key external resource, self-efficacy may function as a critical internal driver, and self-regulated learning is viewed as the core regulatory mechanism. Drawing on integrated theoretical perspectives, we propose that these three elements interact to form a hypothesized motivational process influencing piano learning outcomes. Simultaneously, students’ value orientations, particularly a mastery goal orientation (which focuses on knowledge acquisition, skill improvement, and competency development, rather than merely outperforming others or achieving grades), is recognized as a critical individual difference variable that moderates learning motivation and behavior (Elliot and Hulleman, 2017; Tse et al., 2021).

While existing research in general education has well established the positive roles of teacher support (Stroet et al., 2013), self-efficacy (Usher and Pajares, 2008), and mastery goals (Senko, 2019) for learning outcomes, their integrated function within the specific, high-stakes context of piano instruction in teacher-training universities remains underexplored. Specifically, there is a lack of systematic empirical research that (a) examines how teacher support translates into improved self-regulated learning in piano studies, (b) tests self-efficacy as a potential mediating mechanism in this relationship, and (c) investigates whether a mastery goal orientation serves as a critical boundary condition that strengthens or enables this entire process. This study aims to systematically elucidate these intrinsic mechanisms within the context of piano instruction. The core empirical contribution lies in testing a novel integrated model that combines these constructs, addressing the identified gap. The theoretical contributions are as follows: (1) To apply and specify Bandura’s (1997) concept of triadic reciprocal determinism within the piano learning context. This research integrates teacher support (environmental factor), self-efficacy (personal/cognitive factor), and self-regulated learning (behavioral outcome) within a unified framework, thereby examining a context-specific “environment-person-behavior” triadic mechanism that drives piano learning. (2) To reveal the key mediating pathway of self-efficacy. (3) To pinpoint the moderating role of mastery goals within this triadic system, thereby enriching the application of Achievement Goal Theory in the domain of music skill learning. (4) To bridge macro theory and micro practice by applying and testing an integrated perspective that draws on core tenets from Social Cognitive Theory (Bandura, 1997; emphasizing the interaction of environment, individual cognition, and behavior), Self-Determination Theory (Ryan and Deci, 2020; highlighting the role of autonomy, competence, and relatedness support in intrinsic motivation), and Achievement Goal Theory (Elliot and Hulleman, 2017; specifying the conditional role of mastery goals). This provides a refined theoretical lens for investigating learning motivation in this field.

In summary, by systematically analyzing the interrelationships among teacher support, self-efficacy, and self-regulated learning, and by elucidating the moderating role of mastery goals, this study aims to address a identified research gap and holds significant theoretical innovation value and practical guidance significance. It contributes to a deeper understanding of the endogenous motivational mechanisms in piano learning (and musical skill acquisition more broadly), addresses the challenge of integrating technical training with artistic character development, and ultimately aims to optimize piano teaching practices in higher music teacher education. It is expected that the findings may provide a theoretical foundation and practical insights for informing instructional strategies aimed at stimulating student autonomy and enhancing their capacity for lifelong musical learning and development.

## 2 Theoretical analysis and research hypotheses

The core mission of higher education is to enhance students’ comprehensive qualities and professional competencies. Academic achievement, as a key indicator of educational effectiveness, has seen its underlying influencing factors garner widespread attention from scholars globally. Research has shown that self-regulated learning is a crucial predictor of students’ academic success (e.g., Sitzmann and Ely, 2011; Zimmerman, 2002). The concept of self-regulated learning was first proposed by Zimmerman, referring to the process wherein learners employ cognitive and motivational regulation strategies, autonomously select suitable learning methods, and proactively create supportive learning environments (Zimmerman, 1989). Self-regulated learning is particularly vital in piano studies for music majors in teacher-training universities. This is because achieving proficiency in piano, particularly within a formal curriculum, is recognized to encompass multiple demanding dimensions. These typically include the incremental acquisition of refined techniques, the interpretation of complex repertoire, the cultivation of artistic expressiveness, and the management of performance-related anxiety—challenges that collectively necessitate a high degree of self-regulation. To effectively navigate these multifaceted demands, learners must engage in self-regulatory processes such as hierarchical goal setting, flexible strategy adjustment, and dynamic metacognitive monitoring (Niemiec and Ryan, 2009). Research on self-regulated learning broadly indicates that it is a complex process arising from the synergistic interplay of internal and external factors (Ifenthaler, 2012). This general understanding extends to and underscores the complexity of self-regulated learning within domain-specific contexts such as piano learning for music majors.

The present study is grounded in an integrated theoretical framework that synthesizes key tenets from Social Cognitive Theory (SCT), Self-Determination Theory (SDT), and Achievement Goal Theory (AGT). This integrated perspective offers a coherent system to explain the mechanisms linking teacher support, self-efficacy, and self-regulated learning in piano instruction. Central to this framework is Bandura’s (1997) concept of triadic reciprocal determinism, which posits a dynamic, interdependent relationship between personal factors (e.g., beliefs like self-efficacy), environmental factors (e.g., teacher support), and behavioral outcomes (e.g., SRL). Within this triadic system, teacher support functions as a critical environmental determinant. Drawing on SDT (Ryan and Deci, 2020), particularly its focus on basic psychological needs, teacher support that fosters students’ feelings of autonomy, competence, and relatedness is hypothesized to enhance their intrinsic motivation and personal agency (Ryan and Deci, 2020). This motivational mechanism is crucial for understanding how supportive teaching enhances the personal factor of self-efficacy—a core construct within SCT representing an individual’s belief in their capability to succeed. In turn, heightened self-efficacy is posited to drive the behavioral outcome of more proactive and effective SRL (Zimmerman, 2002). Finally, AGT (Elliot and Hulleman, 2017) specifies a critical boundary condition within this model. It proposes that the strength of the relationships within this triadic system depends on students’ achievement goal orientations. Specifically, a mastery goal orientation, which focuses on developing competence and personal improvement, is expected to strengthen the positive internalization of teacher support into enhanced self-efficacy and, subsequently, more robust SRL behaviors. This integrated framework thus moves beyond viewing the three theories in parallel and instead positions them as complementary lenses explaining the environmental, personal, behavioral, and conditional aspects of the motivational process in piano learning.

Among the various external factors influencing SRL, Teacher Support plays a critical driving role (Stroet et al., 2013; Yin and Xu, 2016). Teacher support is typically defined as a series of supportive behaviors provided by teachers to facilitate student learning and development, encompassing academic, personal, and emotional aspects. In line with the conceptualization used in this study, teacher support is operationalized as encompassing competence support, autonomy support, and emotional support (Chen, 2022). These dimensions manifest in piano instruction as instrumental guidance (e.g., technical demonstration), knowledge facilitation (e.g., stylistic analysis), and emotional care (e.g., encouragement) (Gaunt, 2010). In the specific context of piano instruction in teacher-training universities, the pedagogical approach is characterized by intensive, individualized interaction. This context heightens the potential impact of teacher support on learners’ cognitive regulation, emotional state, and motivation (Zhu et al., 2024). Grounded in Social Cognitive Theory, which emphasizes the influence of environmental factors on individual cognition and behavior, and supported by evidence synthesized in existing reviews linking teacher support to SRL (e.g., Stroet et al., 2013), this study proposes:

*H1*: Teacher support significantly and positively predicts self-regulated learning among piano learners.

Building upon existing research, further exploration of the internal mechanism through which teacher support influences SRL necessitates a focus on Self-efficacy as a key variable. According to Bandura’s (1997) Social Cognitive Theory, self-efficacy is an individual’s belief, confidence, and expectation in their capability to successfully execute the actions required to manage specific situations. It not only directly predicts behavioral outcomes but also plays a central role in self-regulatory processes such as choice of activities, effort expenditure, and persistence. Applied to the domain of piano learning, self-efficacy primarily manifests as the learner’s belief in their ability to overcome technical difficulties, complete specific pieces, achieve precise artistic expression, and perform successfully. Piano learners with high self-efficacy tend to set challenging goals, employ more effective learning strategies (including metacognitive monitoring), invest more time in high-quality practice, and demonstrate greater resilience in overcoming difficulties and setbacks encountered during learning (Pajares, 2008). Bandura (1997) indicated that the formation and development of self-efficacy are influenced by multiple sources, including mastery experiences (personal performance accomplishments), vicarious experiences (e.g., observing teacher demonstrations), verbal persuasion (e.g., positive feedback and high expectations from teachers), and emotional/physiological states (e.g., the positive emotional climate fostered by teacher support). This framework has been widely validated and applied in educational research to understand how teacher behaviors, among other factors, shape student self-efficacy (e.g., Usher and Pajares, 2008). Substantial research confirms that in classroom settings, teacher-provided encouragement and evaluative feedback--conceptualized as critical sources of social persuasion—are key external forces for enhancing students’ academic self-efficacy (Usher and Pajares, 2008; Chai et al., 2011). By fulfilling students’ basic psychological needs for competence, autonomy, and relatedness (Alivernini and Lucidi, 2011), teacher support can significantly enhance their self-efficacy, thereby motivating more proactive and effective self-regulated learning. While self-efficacy itself is recognized as a pivotal construct in piano education (e.g., Gong, 2023), direct empirical investigation into its mediating role between teacher support and self-regulated learning within this specific pedagogical context remains limited. Therefore, based on the solid theoretical foundation and cross-disciplinary evidence outlined above, this study posits:

*H2*: Self-efficacy plays a significant mediating role between teacher support and self-regulated learning among piano learners.

Having preliminarily revealed that teacher support may influence SRL through the mediating path of self-efficacy, a deeper question arises: whether this mechanism differs among learners due to individual differences in inherent achievement motivation orientations. Achievement Goal Theory provides a crucial perspective for delving into this issue. This theory distinguishes core dimensions of achievement goals, primarily mastery goals and performance goals. A mastery goal orientation focuses on developing competence, completing tasks, acquiring knowledge, and realizing self-improvement for its intrinsic value. Learners with a high mastery goal orientation are primarily motivated by an inherent interest in the knowledge/skills themselves and a desire for growth (Gou et al., 2024; Senko, 2019). Within the SRL framework, a mastery goal orientation is believed to effectively promote the use of deep learning strategies, enhance self-monitoring and regulatory capacity during learning, and increase perseverance in the face of difficulties (Pintrich, 2000; Senko, 2019). Therefore, this study focuses on the mechanism of mastery goals in the relationship between teacher support and SRL. Therefore, this study focuses on the mechanism of mastery goals in the relationship between teacher support and SRL. Theoretically, for students with a high mastery goal orientation, teacher support behaviors (e.g., encouraging exploration, emphasizing progress, providing constructive feedback rather than simple evaluation) are more likely to be interpreted as “opportunities for growth” and “assistance for skill enhancement,” rather than as “evaluative pressure.” Following this interpretation, such students would be better positioned to actively translate teacher support into adaptive self-learning strategies and maintain stronger intrinsic learning motivation. It is also plausible that the intrinsic drive inherent in a strong mastery goal orientation could, to some extent, compensate for a deficiency in external teacher support, thereby helping to sustain self-regulated learning behaviors (Chen, 2022). Thus, mastery goals may not only directly promote SRL but also potentially moderate the strength of the relationships within the proposed triadic system. Thus, mastery goals may not only directly promote SRL but also potentially moderate the strength of the relationships within the proposed triadic system. Specifically, it is hypothesized to strengthen the positive effect of teacher support on self-efficacy and, consequently, on self-regulated learning. Based on this, the study proposes:

*H3*: Mastery goals play a significant moderating role in the effect of teacher support on self-regulated learning among piano learners.

**Figure 1:**
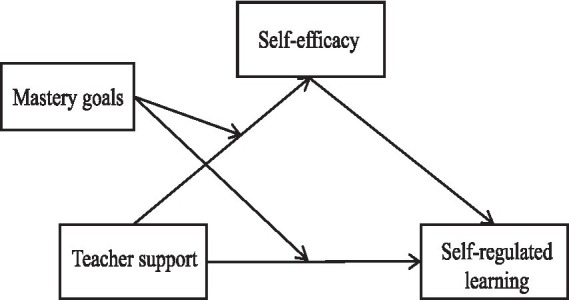
The hypothesized model to be tested in this study.

The overall hypothesized model integrating these relationships (H1, H2, H3) is presented in Figure 1.

## 3 Research methods

### 3.1 Participants

A cluster sampling method was employed to select participants from the music schools of two teacher-training universities in Anhui Province. The “clusters” were intact classes from the first to the fourth year. Classes were selected based on availability and curriculum schedules, aiming to include at least one class from each academic year in each university. This approach ensured the sample represented students across all years of the program while maintaining practicality. A total of 500 questionnaires were distributed. Questionnaires were deemed invalid and excluded (*n* = 36) for the following reasons: uniform responses (*n* = 22), over 10% missing data (*n* = 9), and unreasonably short completion time (*n* = 5). After this, 464 valid questionnaires were retained, yielding an effective response rate of 92.8%. The sample consisted of 101 male students (21.77%) and 363 female students (78.23%). In terms of grade distribution, there were 145 freshmen (31.25%), 120 sophomores (25.86%), 193 juniors (41.59%), and 6 seniors (1.29%). The low number of seniors is because their curriculum focuses more on teaching practicum and thesis work, with less structured piano class attendance. All participants were enrolled in a music education program, which included systematic compulsory piano courses as part of their curriculum.

To evaluate the adequacy of the sample size, a *post hoc* power analysis was performed using G*Power 3.1 software with the number of predictors set to 5 and α = 0.05. The effect size was specified as *f*^2^ = 0.25, which is considered medium according to Cohen’s conventional criteria (Cohen, 1988) and is consistent with the moderate to high intercorrelations observed among the study variables (see Table 1). The analysis revealed a statistical power > 0.99, indicating that the current sample size was sufficient to detect medium or larger effects.

**Table 1:** Descriptive statistics, normality indices, and correlations among variables (*N* = 464).

Variable	*M*	SD	Skewness	SE (Skew)	Kurtosis	SE (Kurt)	1	2	3	4
1. Teacher support	4.33	0.55	−0.43	0.13	−0.58	0.25	1			
2. Self-efficacy	3.64	0.64	0.15	0.13	−0.12	0.25	0.50^**^	1		
3. SRL	3.81	0.54	0.32	0.13	−0.07	0.25	0.50^**^	0.78^**^	1	
4. Mastery goals	4.07	0.63	−0.28	0.13	−0.36	0.25	0.50^**^	0.57^**^	0.73^**^	1

SE, Standard Error. *p < 0.05, **p < 0.01, ***p < 0.001.

### 3.2 Measures

#### 3.2.1 Teacher support scale

Teacher support was measured using the scale revised by Chen (2022). It comprises 13 items that assess three dimensions of support specific to the piano instruction context: competence support (e.g., “In class, my piano teacher explains musical works in detail and demonstrates performance”), autonomy support (e.g., “My piano teacher encourages me to voice my own ideas and listens to them”), and emotional support (e.g., “My piano teacher often gives me encouragement”). Participants rated their agreement on a 5-point Likert scale from 1 (strongly disagree) to 5 (strongly agree). All items are positively worded, with higher composite scores representing a higher overall level of perceived teacher support. In the current sample, the scale demonstrated excellent reliability (Nunnally, 1978). Confirmatory Factor Analysis (CFA) indicated a good model fit (χ^2^/df = 2.81, CFI = 0.96, TLI = 0.95, RMSEA = 0.07, SRMR = 0.05; see Hu and Bentler, 1999, for fit index criteria). The overall Cronbach’s α was 0.95, and the reliability coefficients for the subscales were 0.88 for competence support, 0.90 for autonomy support, and 0.91 for emotional support.

#### 3.2.2 Academic self-efficacy scale

The Academic Self-Efficacy Scale revised by Gong (2023) was adopted. This scale was specifically developed to measure self-efficacy within the domain of piano learning. It contains 22 items covering two dimensions: self-efficacy for learning ability and self-efficacy for learning behavior. Example items include: “I believe I can apply the knowledge I’ve learned to solve practical problems encountered while playing the piano” (ability efficacy), and “I often choose challenging piano pieces from which I can learn, even if it requires more effort” (behavior efficacy). The scale uses a 5-point Likert format, from 1 (strongly disagree) to 5 (strongly agree), where higher scores indicate stronger self-efficacy. Confirmatory Factor Analysis (CFA) supported the construct validity of the scale in the present study (χ^2^/df = 3.12, CFI = 0.95, TLI = 0.94, RMSEA = 0.08, SRMR = 0.04). The Cronbach’s α coefficient for this scale in the current study was 0.98, indicating excellent internal consistency reliability.

#### 3.2.3 Self-regulated learning scale

Based on the self-regulated learning questionnaire developed by Schunk and Ertmer (2000), a scale was adapted and translated considering the context of piano instruction. To ensure conceptual equivalence, a rigorous translation-back-translation procedure was followed: the original scale was independently translated by two researchers and synthesized into a Chinese draft; this draft was then back-translated by a third translator unfamiliar with the original version; finally, the research team and a piano education expert compared the original, translated, and back-translated versions, reviewing and making contextual adjustments to item wording. The final scale consists of 16 items assessing students’ self-regulated learning across multiple dimensions, including motivation control, learning methods, strategy implementation, and the effective use of environmental resources. It measures key self-regulated learning processes such as goal setting (e.g., “In class, I set specific goals for my piano learning”), strategy adjustment (e.g., “If I find my learning method ineffective, I improve it”), and self-monitoring (e.g., “I refer to previously set goals to measure my progress”). The questionnaire employs a 5-point Likert scale from 1 (strongly disagree) to 5 (strongly agree), with higher scores indicating stronger self-regulated learning in piano studies. Confirmatory Factor Analysis (CFA) indicated a good model fit (χ^2^/df = 2.95, CFI = 0.96, TLI = 0.95, RMSEA = 0.07, SRMR = 0.06). The Cronbach’s α coefficient for this scale in the present study was 0.96.

#### 3.2.4 Mastery goals scale

The mastery goals subscale from the Achievement Goal Questionnaire developed by Elliot and Murayama (2008) was used. It contains 6 items (e.g., “I want to learn as much as possible from this course” and “In class, I prefer challenging material from which I can learn new things”) and employs a 5-point Likert scale ranging from 1 (strongly disagree) to 5 (strongly agree). Higher scores indicate a stronger tendency toward adopting mastery goals. All items are positively worded, assessing the focus on learning and personal improvement. Confirmatory Factor Analysis (CFA) demonstrated a good fit and supported the structural validity of the scale in this study (χ^2^/df = 3.25, CFI = 0.94, TLI = 0.93, RMSEA = 0.07, SRMR = 0.04). In this study, the Cronbach’s α coefficient for this questionnaire was 0.95.

### 3.3 Analytical procedures

Data processing and statistical analyses were conducted using SPSS 26.0 and AMOS 26.0. First, structural equation modeling (SEM) was employed as the primary analytical framework, utilizing the maximum likelihood (ML) estimator in AMOS 26.0. The choice of ML estimation was justified as the univariate skewness and kurtosis values for all observed variables fell within acceptable thresholds (see Table 1), and no severe multivariate outliers were detected, thus providing reasonable assurance that the assumption of multivariate normality was not severely violated (Kline, 2016). Initial confirmatory factor analysis (CFA) was performed to assess the validity of the measurement models. To control for common method bias, procedural remedies--including anonymous responses and counterbalancing the order of scale items--were implemented during data collection (Podsakoff et al., 2003). *Post hoc*, Harman’s single-factor test was conducted to statistically evaluate potential bias (Fuller et al., 2016; Podsakoff et al., 2003). Descriptive statistics and correlation analyses were then performed using SPSS to gain a preliminary understanding of data distribution characteristics and the relationships among variables.

Building on this foundation and guided by relevant theories and research hypotheses, a mediation model was tested to examine the role of self-efficacy. Specifically, Model 4 from the PROCESS macro developed by Hayes (2018) was employed to conduct a Bootstrap mediation analysis. To test the moderating effect of mastery goals, Model 8 of the PROCESS macro was used to analyze a moderated model. This model allows for the simultaneous examination of mastery goals as a moderator in two paths: (1) moderating the effect of teacher support on self-efficacy (the mediator), and (2) moderating the direct effect of teacher support on self-regulated learning (the outcome variable). All analyses were conducted while controlling for gender and academic year, with the aim of systematically investigating the mechanism through which teacher support influences self-regulated learning in piano students, as well as the roles played by self-efficacy and mastery goals within this mechanism.

## 4 Results

### 4.1 Tests for common method bias

*Post hoc*, a Harman‘s single-factor test was conducted to systematically assess its potential impact (Podsakoff et al., 2003). Following the standard procedure, all items from the four main constructs (teacher support, self-efficacy, self-regulated learning, mastery goals) were entered into an unrotated exploratory factor analysis using principal component analysis. The results indicated that five factors had eigenvalues greater than 1. The first factor accounted for 33.04% of the total variance, which is below the commonly applied heuristic threshold of 40% (e.g., Podsakoff et al., 2003; West et al., 1995). While acknowledging the diagnostic limitations of this test, this result provides preliminary evidence that common method bias is unlikely to be a severe threat to the validity of our findings.

### 4.2 Difference analysis of self-regulated learning across demographic variables

To examine the influence of gender and academic year on the study variables, difference tests were conducted separately. The results of an independent-samples t-test showed no significant difference in self-regulated learning levels between students of different genders (*t* = 0.73, *p* = 0.47). The self-regulated learning levels of male students (*M* = 3.85, SD = 0.59) and female students (*M* = 3.80, SD = 0.52) were comparable.

A one-way analysis of variance (ANOVA) was performed after confirming the assumption of homogeneity of variances (Levene’s test, *p* = 0.73). The results indicated that there was no statistically significant difference in self-regulated learning levels across academic years (*F* = 1.58, *p* = 0.19, η^2^ = 0.01; indicating a negligible effect size according to conventional benchmarks, Cohen, 1988). This suggests that students’ self-regulated learning remained stable across different academic years and did not change significantly with the progression of their studies.

Based on the above analyses, and to control for the potential confounding influence of these demographic variables and thereby enhance the rigor of the research conclusions, gender and academic year were still included as control variables in all subsequent mediation and moderation analyses.

### 4.3 Descriptive statistics and correlation analysis

The descriptive statistics and intercorrelations for the study variables are presented in Table 1. As shown, all variables were significantly and positively intercorrelated (all *p* values < 0.01), with magnitudes ranging from moderate to large according to conventional benchmarks (Cohen, 1988). This pattern, particularly the strong correlation between self-efficacy and self-regulated learning (*r* = 0.78), provides initial support for the relationships hypothesized in our integrated model and justifies the subsequent mediation and moderation analyses. A brief interpretation of the mean scores suggests that participants, on average, perceived a high level of teacher support (*M* = 4.33) and strongly endorsed a mastery goals (*M* = 4.07). Self-efficacy (*M* = 3.64) and self-regulated learning (*M* = 3.81) were also reported at moderately high levels, all clearly above the scale midpoint of 3 on the 5-point Likert scale. Univariate normality was assessed through skewness and kurtosis statistics. For teacher support, skewness was −0.43 (SE = 0.13) and kurtosis was −0.58 (SE = 0.25); for self-efficacy, skewness was 0.15 (SE = 0.13) and kurtosis was −0.12 (SE = 0.25); for self-regulated learning, skewness was 0.32 (SE = 0.13) and kurtosis was −0.07 (SE = 0.25); and for mastery goals, skewness was −0.28 (SE = 0.13) and kurtosis was −0.36 (SE = 0.25). All skewness and kurtosis values fell within the commonly recommended thresholds for normal distribution (i.e., absolute skewness < 2 and absolute kurtosis < 7), indicating no severe violations of univariate normality (Kline, 2016). Together with the absence of severe multivariate outliers (as assessed by examining Mahalanobis distances), these diagnostics provided reasonable assurance that the assumption of multivariate normality was not severely violated, supporting the use of maximum likelihood estimation in the initial confirmatory factor analyses and overall model fit evaluation (Kline, 2016).

### 4.4 Mediating effect of self-efficacy

Prior to testing the specific mediation hypothesis, the overall measurement model was evaluated using structural equation modeling (SEM). The model demonstrated an acceptable fit according to conventional criteria (Hu and Bentler, 1999): χ^2^/df = 2.87, CFI = 0.96, TLI = 0.95, RMSEA = 0.07, SRMR = 0.04, supporting the tenability of the proposed relationships.

To further investigate the mechanism through which teacher support influences self-regulated learning, a mediation analysis was conducted with teacher support as the independent variable, self-efficacy as the mediator, and self-regulated learning as the outcome variable, while controlling for gender and academic year. Gender was coded as 0 = male, 1 = female. Academic year was coded from 1 to 4, representing freshman to senior year. Model 4 from Hayes’s SPSS PROCESS macro was utilized, employing a bootstrap sampling method with 5,000 resamples and a 95% confidence interval. The results are presented in Tables 2, 3. It should be noted that Table 2 reports the standardized coefficients (β) for the specific paths of the mediation model, while Table 3 presents the bootstrap-estimated standardized effect sizes (i.e., total, direct, and indirect effects) which are central to testing our hypotheses. These two tables provide complementary information: the path coefficients (Table 2) depict the relationships within the model, and the effect sizes (Table 3) offer robust estimates for the effects of interest. The slight numerical differences between the standardized path coefficients (β) in Table 2 and the bootstrap-estimated effect sizes in Table 3 are expected, as the former are point estimates from regression, while the latter are central estimates (e.g., means) derived from the bootstrap sampling distribution.

**Table 2:** Analysis of the mediating effect of self-efficacy between teacher support and self-regulated learning.

Predictor	DV: self-regulated learning			DV: self-efficacy (Step 1)			DV: self-regulated learning (Step 2)		
	β	SE	*t*	β	SE	*t*	β	SE	*t*
Teacher support	0.50	0.04	11.05^***^	0.50	0.05	10.99^***^	0.15	0.04	3.99^***^
Self-efficacy							0.71	0.03	19.66^***^
Gender	−0.02	0.06	−0.34	−0.09	0.07	−1.97	0.05	0.04	1.50
Academic year	−0.02	0.03	−0.37	0.04	0.03	0.85	−0.04	0.02	−1.39
*R*^2^	0.25			0.25			0.63		
*F*	42.59^***^			42.42^***^			161.38^***^		

DV, Dependent Variable. *p < 0.05, **p < 0.01, ***p < 0.001.

**Table 3:** Bootstrap estimates of standardized effects for the mediation model.

Effect type	Effect size	Boot SE	Bootstrap 95% CI		Relative effect
			Lower	Upper	
Total effect	0.49	0.04	0.40	0.57	
Direct effect	0.14	0.04	0.07	0.21	29.10%
Indirect effect	0.34	0.04	0.28	0.42	70.90%

**Figure 2:**
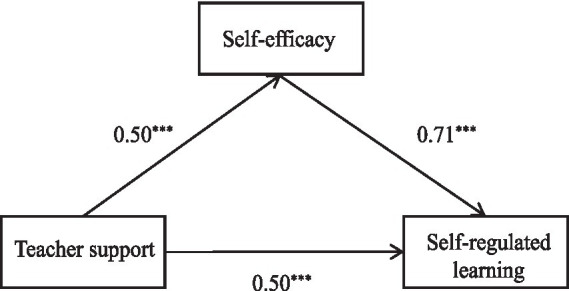
Mediation model of self-efficacy between teacher support and self-regulated learning. *** denote the standardized path coefficients that are statistically significant at *p* < 0.001.

The regression results in Table 2 (first column) indicate that teacher support had a significant positive total effect on self-regulated learning before accounting for the mediator (β = 0.50, *p* < 0.001), providing initial support for Hypothesis 1. Concurrently, teacher support also significantly and positively influenced self-efficacy (β = 0.50, *p* < 0.001). After controlling for teacher support, self-efficacy remained a significant positive predictor of self-regulated learning (β = 0.71, *p* < 0.001). When self-efficacy was included in the model, the direct effect of teacher support on self-regulated learning remained significant (β = 0.15, *p* < 0.001). Notably, the inclusion of self-efficacy substantially increased the explained variance in self-regulated learning from 25% (Step 1) to 63% (Step 2). The model for the mediator (self-efficacy) explained 25% of the variance. The coefficients for the covariates (gender, academic year) were not statistically significant in these models. The standardized path coefficients from this PROCESS analysis are presented in Figure 2.

According to the bootstrap test results for the mediation effect presented in Table 3 (95% CI), teacher support had a significant total effect on self-regulated learning (total effect = 0.49). Within this, the direct effect was 0.14 (accounting for 29.10% of the total effect), and the indirect effect mediated through self-efficacy was 0.34 (accounting for 70.90% of the total effect). The confidence interval for the indirect effect (0.28, 0.42) did not include zero, indicating that self-efficacy played a significant partial mediating role in the relationship between teacher support and self-regulated learning. Therefore, Hypothesis 2 was supported.

### 4.5 Moderating effect of mastery goals

Model 8 from the PROCESS macro was employed to test the moderated model while controlling for gender and academic year. Prior to analysis, the variables of teacher support and mastery goal orientation were mean-centered. The results are presented in Table 4.

**Table 4:** Analysis of the moderating effect of mastery goal orientation.

Predictor	DV: self-efficacy			DV: self-regulated learning		
	β	SE	*t*	β	SE	*t*
Teacher support	0.36	0.06	6.57^*^	0.07	0.03	2.23^*^
Self-efficacy				0.43	0.03	14.99^***^
Mastery goals	0.45	0.045	9.41^*^	0.35	0.03	11.64^*^
TS × MG	0.17	0.07	2.53^*^	0.14	0.04	3.69^***^
Gender	−0.16	0.06	−2.49^*^	0.03	0.04	0.75
Academic year	0.08	0.03	2.78^**^	0.02	0.02	1.21
*R*^2^	0.41			0.74		
*F*	52.12^***^			175.64^***^		

Prior to analysis, the continuous variables of “Teacher Support” and “Mastery Goal Orientation” were mean-centered. The variance inflation factor (VIF) for all independent variables was below 2, indicating no severe multicollinearity issues. TS, Teacher Support; MG, Mastery Goals; DV, Dependent Variable. *p < 0.05, **p < 0.01, ***p < 0.001.

Analysis of the moderated results (Table 4) reveals the following pattern: First, regarding the prediction of self-efficacy, teacher support had a significant positive main effect (β = 0.36, *p* < 0.05). Mastery goals also demonstrated a significant positive main effect on self-efficacy (β = 0.45, *p* < 0.05), indicating that students with a stronger focus on learning and mastery tend to report higher beliefs in their piano learning capabilities. Crucially, the interaction between teacher support and mastery goals was also significant (β = 0.17, *p* < 0.05). In regression models containing a significant interaction term, the main effect represents the relationship when the moderator (mastery goals) is at its average level. This significant interaction indicates that the strength of the relationship between teacher support and self-efficacy varies systematically depending on students’ levels of mastery goals, aligning with Achievement Goal Theory which posits that a mastery orientation enhances the internalization of external resources (Elliot and Hulleman, 2017). Second, when self-regulated learning was the outcome, teacher support (β = 0.07, *p* < 0.05), self-efficacy (β = 0.43, *p* < 0.001), mastery goals (β = 0.35, *p* < 0.001), and their interaction term (β = 0.14, *p* < 0.001) were all significant predictors. In summary, the results indicate that mastery goals significantly moderate the effects of teacher support on both self-efficacy and self-regulated learning. This finding provides direct support for Hypothesis 3, which postulated that mastery goal orientation moderates the relationship between teacher support and self-regulated learning among piano learners.

**Figure 3:**
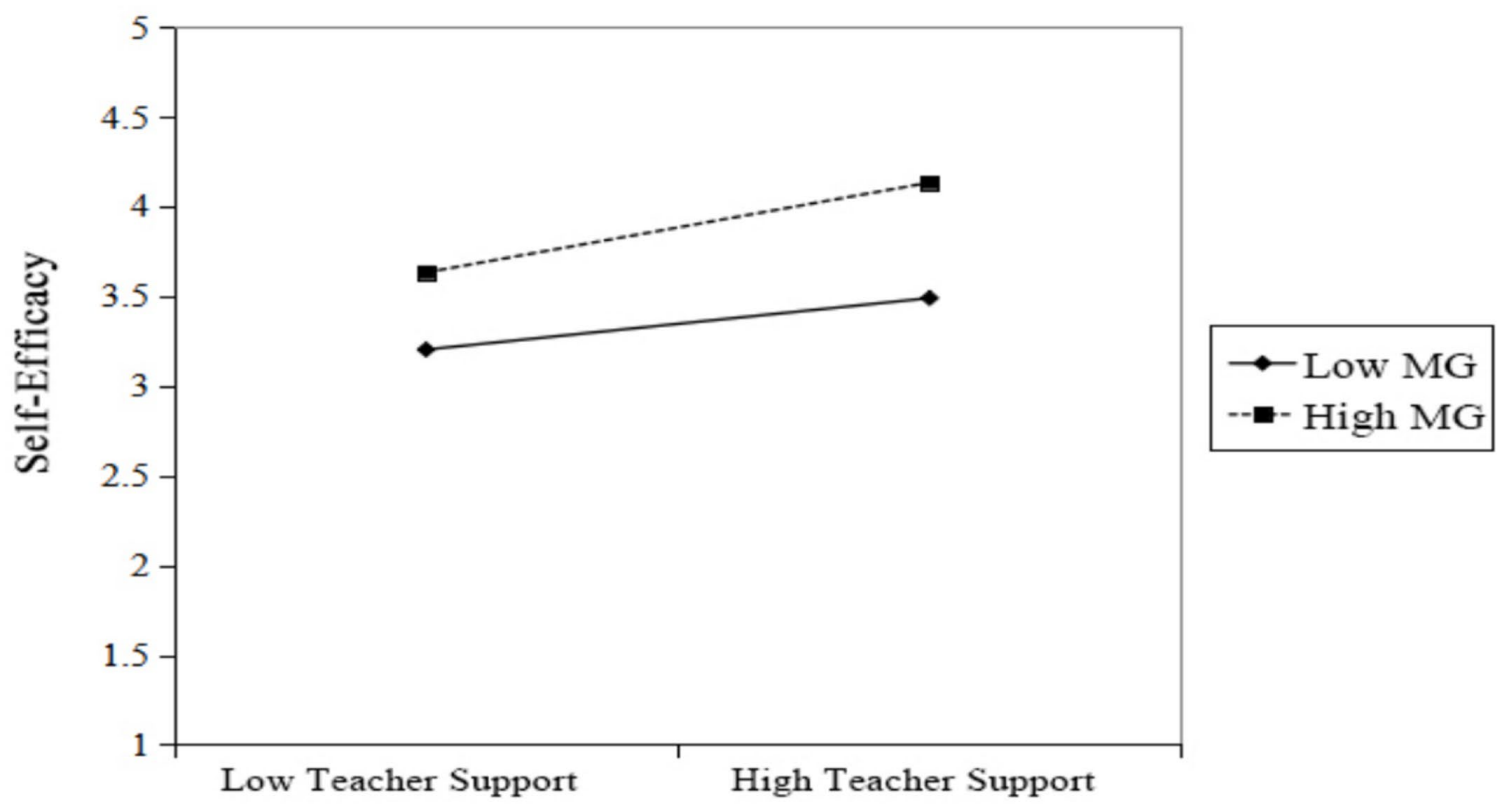
Simple slopes for the moderation of teacher support on self-efficacy by mastery goals.

**Figure 4:**
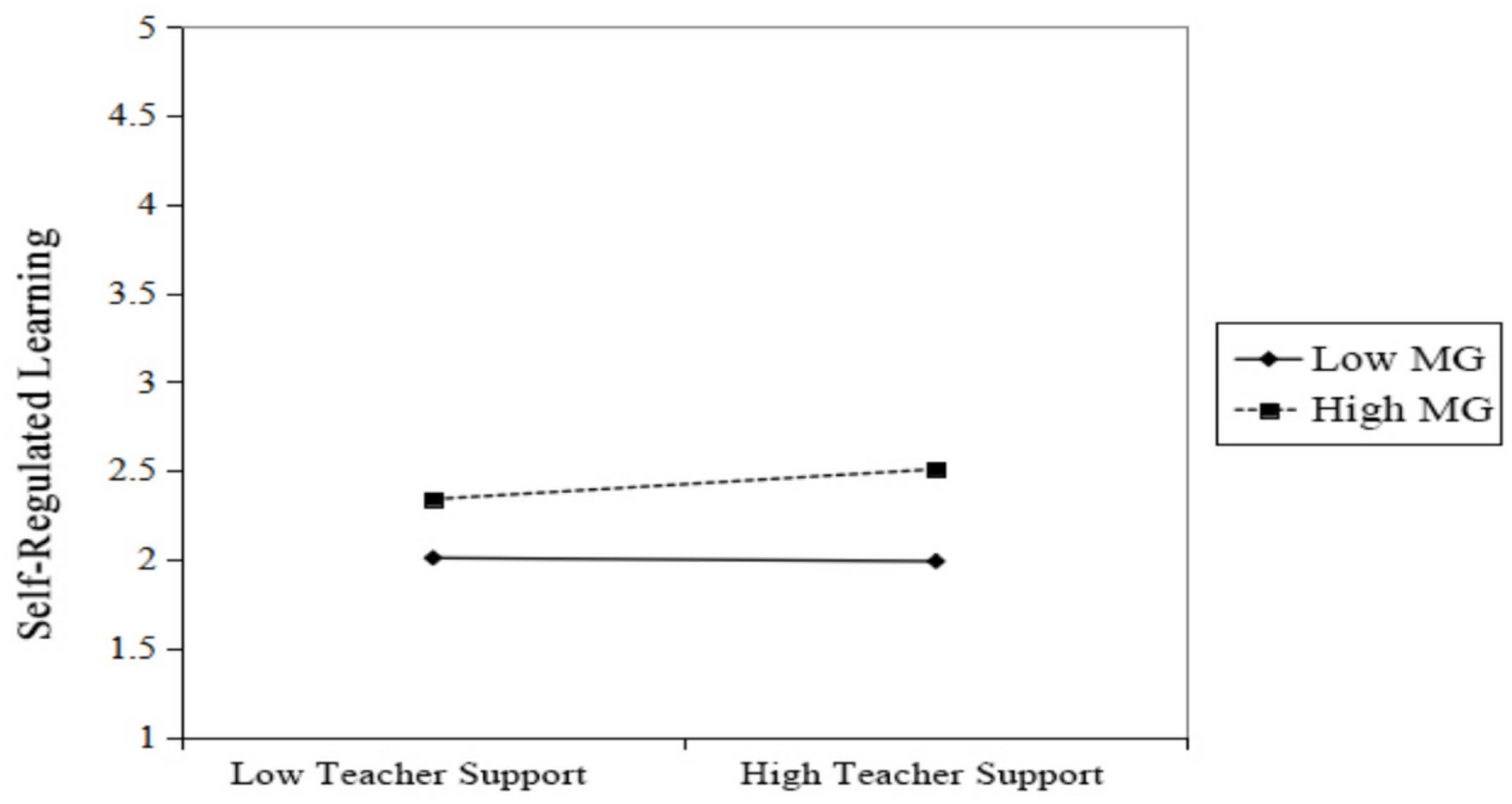
Simple slopes for the moderation of teacher support on self-regulated learning by mastery goals.

To further elucidate the nature of the moderating effect, we probed the simple slopes at specific levels of the continuous moderator (mastery goals). Specifically, the conditional effects of teacher support were examined at values corresponding to one standard deviation above and below the mean of mastery goals (as shown in Figures 3, 4). As seen in Figure 3, teacher support had a positive predictive effect on self-efficacy regardless of students’ mastery goals levels (Low Mastery Goals: β = 0.26, *p* < 0.001; High Mastery Goals: β = 0.47, *p* < 0.001). The strength of this positive relationship increased with higher levels of mastery goals, indicating that mastery goals significantly moderate the relationship between teacher support and self-efficacy.

The results in Figure 4 show that when students had high levels of mastery goals, teacher support significantly and positively predicted their self-regulated learning (β = 0.16, *p* < 0.001). Conversely, for students with low mastery goal levels, teacher support was not a significant predictor of self-regulated learning (β = −0.01, *p* = 0.68). Therefore, mastery goals significantly moderated the relationships between teacher support and self-efficacy, as well as between teacher support and self-regulated learning.

## 5 Discussion

Grounded in an integrated theoretical perspective that draws on Self-Determination Theory (emphasizing the role of teacher support), Social Cognitive Theory (emphasizing self-efficacy and self-regulation), and Achievement Goal Theory (specifying the conditional role of mastery goals), this study delved into the mechanisms linking teacher support, self-efficacy, mastery goals, and self-regulated learning within piano instruction for music majors at teacher-training universities. The empirical results provided support for the proposed hypotheses, suggesting an internal pathway and boundary conditions through which teacher support is associated with college students’ self-regulated learning in piano. These findings offer preliminary empirical insights that may inform efforts to optimize piano pedagogy in higher teacher education institutions.

### 5.1 The mediating role of self-efficacy between teacher support and self-regulated learning in piano learners

First, this study found a significant positive correlation between teacher support and piano learners’ self-regulated learning (SRL). This finding is consistent with the applicability of Self-Determination Theory and Social Cognitive Theory in the field of music education. This direct association not only aligns with established findings on the positive role of need-supportive teaching for student motivation and engagement in general education (Stroet et al., 2013) but also resonates with the recognized importance of positive teacher-student dynamics in music learning contexts. For instance, supportive relationships are considered foundational for fostering student agency and adaptive learning in higher education settings, including those involving skill acquisition (Jääskelä et al., 2017). Our study extends this line of inquiry by quantitatively linking multifaceted teacher support to the specific outcome of SRL within the domain of piano instruction at teacher-training universities. In line with the conceptualization used in this study (Chen, 2022), teacher support refers to systematic support for student autonomy, competence, and emotional needs. Within the apprenticeship-like context of piano instruction, supportive behaviors from teachers (e.g., instrumental guidance, emotional care, autonomy respect) may help create a learning environment that facilitates strategic modeling and psychological safety, factors conducive to SRL (Chen and Guo, 2016; Gaunt, 2010). Such an environment may encourage students to engage in experimentation and reflection, thereby supporting their use of self-regulatory strategies to manage challenges, such as those presented by demanding repertoire (Wang, 2024). These associations provide preliminary evidence that could inform efforts to enhance teacher-student interaction and support strategies in piano pedagogy at teacher-training institutions.

Second, and more importantly, this study found that self-efficacy played a significant partial mediating role in the relationship between teacher support and SRL. This finding suggests a potential “external support → internal belief → self-regulated behavior” pathway. This proposed pathway offers empirical support that is consistent with the core proposition of Social Cognitive Theory regarding the dynamic interplay between environment, personal factors, and behavior (Bandura, 1997). Moreover, it aligns with the established understanding from general education that teacher behaviors (a key source of social persuasion) are significant antecedents of academic self-efficacy (Usher and Pajares, 2008), which in turn is a robust predictor of adaptive learning processes. By testing this mediation model in the context of piano education, our findings address a relative gap in music education literature, where the specific mechanisms linking teacher support to SRL via self-efficacy have received limited direct empirical attention (Gu and Zhang, 2024). More specifically, within the context of piano instruction for future music teachers, our study provides initial empirical support for the proposition that teacher support is associated with SRL not only directly but also indirectly through its positive association with students’ self-efficacy. Theoretically, effective teacher support—such as competence feedback, autonomy-respectful instruction, and emotional encouragement—may help strengthen students’ belief in their capability to master piano skills. Our data indicate that higher self-efficacy is associated with more frequent and proactive SRL behaviors, such as setting challenging goals, employing metacognitive strategies, and persisting in practice (Lu et al., 2022). This pattern suggests that a key function of teacher support in piano pedagogy may lie in its potential to empower students by fostering the self-belief that they can succeed, which may in turn motivate and sustain more self-regulated engagement in learning.

### 5.2 The moderating role of mastery goals in the relationship between teacher support and self-regulated learning

The empirical findings further indicate a significant moderating role of mastery goals in the process linking teacher support to college students’ self-regulated learning in piano, which is consistent with and extends the applicability of Achievement Goal Theory in the field of music education. Specifically, the identification of mastery goals as a significant moderator in both paths of our model provides nuanced empirical evidence for Achievement Goal Theory’s premise that goal orientations shape how individuals engage with and benefit from their learning environments (Elliot and Hulleman, 2017). The moderating effect of mastery goals is evident at two levels (corresponding to Figures 3, 4, respectively). In statistical terms, the positive interaction effects signify that the beneficial relationship between teacher support and student outcomes is amplified among students who more strongly endorse mastery goals.

First, mastery goals moderate the relationship between teacher support and self-efficacy (see Figure 3). For students with high mastery goals, they are more likely to perceive a teacher’s technical guidance and encouraging feedback as valuable “resources for growth” and “opportunities for skill enhancement.” This aligns with the view that a mastery goal orientation reflects a focus on developing competence and personal growth (Senko, 2019). Learners with such an orientation may therefore be more inclined to engage with and value teacher support as a resource for learning. This positive cognitive interpretation enables them to derive confidence more effectively from teacher support, thereby significantly boosting their self-efficacy for overcoming technical challenges. Simple slope analysis shows that as students’ mastery goal level increases, the facilitative effect of teacher support on self-efficacy strengthens significantly (see Figure 3). This explains why students with high mastery goals often exhibit greater confidence and resilience when facing difficulties.

Second, mastery goals also moderate the direct effect of teacher support on self-regulated learning (see Figure 4). When students hold high mastery goals, teacher support can be more effectively translated into direct motivation for them to set practice goals, monitor progress, and adjust strategies. Conversely, for students with low levels of mastery goals, teacher support is less effective in directly stimulating their deeper self-regulated learning behaviors. Taken together, these interaction effects not only align with achievement goal theory (Elliot and Hulleman, 2017) but also corroborate empirical findings on the conditional role of mastery goals (e.g., Huang, 2012; Senko and Harackiewicz, 2002). From this perspective, mastery goals may shape the appraisal and utilization of teacher support, thereby enhancing its translation into both stronger self-efficacy beliefs and more active self-regulated learning behaviors. Collectively, these findings suggest that students’ mastery goal orientation may serve as an important boundary condition that influences the strength of the associations between teacher support and both self-efficacy and self-regulated learning. It specifies that the positive influence of teacher support on SRL is contingent upon students’ pre-existing focus on growth and mastery, thereby clarifying when and for whom teacher support is most potent.

For piano pedagogy in teacher-training universities, these findings highlight a key consideration. They suggest that merely enhancing the quality and frequency of teacher support may not be sufficient, by itself, to ensure the effective improvement of students’ self-regulatory abilities. Pedagogical efforts may therefore need to also cultivate and stimulate students’ value orientation toward pursuing high mastery goals, to create an environment where teacher support is more consistently associated with the development of self-regulated learning.

### 5.3 Implications and limitations

By examining the mechanisms linking teacher support, self-efficacy, mastery goals, and self-regulated learning, this study provides theoretical support and practical implications for piano instruction in higher music education institutions.

At the theoretical level, the primary contribution of this study lies in revealing the unique moderating role of “mastery goals” in the piano learning context through a moderated model. The results demonstrate that mastery goals not only enhance the facilitative effect of teacher support on self-efficacy but also strengthen its direct effect on SRL. This offers a concrete empirical case for integrating Self-Determination Theory (emphasizing support), Social Cognitive Theory (emphasizing efficacy and regulation), and Achievement Goal Theory (emphasizing goal orientation), clarifying the specific pathway through which external support influences learning behaviors via the combined effects of individual cognition and motivational dispositions.

At the practical level, the findings offer insights that may inform piano pedagogy. Based on the observed associations among perceived teacher support, self-efficacy, mastery goals, and self-regulated learning (SRL), we suggest the following pedagogical implications that are consistent with these correlational patterns. The results suggest that fostering self-regulated learning effectively could involve a dual focus. First, it may be beneficial for teachers to construct a multidimensional support system encompassing competence support (e.g., targeted technical guidance), autonomy support (e.g., offering choices in repertoire interpretation), and emotional support that emphasizes personal progress. Second, parallel efforts could focus on cultivating a classroom climate conducive to mastery goals. This can be achieved through task design (e.g., setting individualized, improvement-based challenges) and feedback practices (e.g., praising effort and strategic process). By integrating supportive teaching with the promotion of a mastery orientation, instructors may create conditions where teacher support is more consistently and strongly associated with the development of students’ self-efficacy and self-regulated learning behaviors. It is important to note that these suggested approaches, derived from the current correlational findings, would benefit from further validation through longitudinal or intervention studies to establish their causal efficacy.

This study also has several limitations. First, the cross-sectional design precludes firm conclusions regarding the causal direction between the variables. Second, as the sample was drawn exclusively from music education majors in teacher-training universities, the generalizability of the findings to students in conservatories or comprehensive universities requires further validation. Third, although rigorous translation-back-translation procedures were followed for the Self-Regulated Learning Scale, detailed information regarding its adaptation and validation within the unique context of piano instruction at Chinese teacher-training universities remains limited. Future studies could provide more robust evidence for the contextual validity of this measure. Finally, all main variables were measured using self-report methods; future research could incorporate more objective indicators such as teacher assessments or behavioral observations. Building on this study’s emphasis on the distinctive context of Chinese normal universities and their apprenticeship-style piano instruction, we explicitly propose these contextual characteristics as key foci for future cross-cultural or multi-context comparative research. Future studies employing longitudinal designs or intervention experiments would also be valuable to further test and apply these findings.

## 6 Conclusion

Based on an empirical investigation of music education majors at teacher-training universities, this study examined the relationships among teacher support, self-efficacy, mastery goals, and self-regulated learning (SRL) in piano instruction. The results indicated the following:

1. Teacher support was positively associated with piano learners’ self-regulated learning, both directly and indirectly through its positive association with self-efficacy. Self-efficacy was found to play a significant partial mediating role in this relationship.
2. Mastery goals significantly moderated the observed associations. Specifically, the positive relationships of teacher support with both self-efficacy and self-regulated learning were stronger among students who reported higher levels of mastery goals.

## Data Availability

The raw data supporting the conclusions of this article will be made available by the authors, without undue reservation.
